# Efficacy of Dose-Titrated Glucagon Infusions in the Management of Congenital Hyperinsulinism: A Case Series

**DOI:** 10.3389/fendo.2020.00441

**Published:** 2020-09-03

**Authors:** Maria Salomon-Estebanez, Daphne Yau, Mark J. Dunne, Chris Worth, Sune Birch, José L. Walewski, Indraneel Banerjee

**Affiliations:** ^1^Department of Paediatric Endocrinology, Royal Manchester Children's Hospital, Manchester, United Kingdom; ^2^Faculty of Biology, Medicine and Health, University of Manchester, Manchester, United Kingdom; ^3^Department of Statistics, Zealand Pharma A/S, Søborg, Denmark; ^4^Medical Publications, rareLife Solutions, Norwalk, CT, United States

**Keywords:** glucose, congenital hyperinsulinism, hypoglycemia, glucagon, infusion, dose titration

## Abstract

**Background:** Congenital hyperinsulinism (CHI), a rare disease of excessive and dysregulated insulin secretion, can lead to prolonged and severe hypoglycemia. Dextrose infusions are a mainstay of therapy to restore normal glycemia, but can be associated with volume overload, especially in infants. By releasing intrahepatic glucose stores, glucagon infusions can reduce dependency on dextrose infusions. Recent studies have reported positive outcomes with glucagon infusions in patients with CHI; however, to date, there are no reports describing the clinical utility of titrated doses of infused glucagon to achieve glycemic stability.

**Objective:** To assess the potential clinical utility of dose-titrated glucagon infusions in stabilizing glycemic status in pediatric patients with CHI, who were managed by medical and/or surgical approaches.

**Methods:** Patients with CHI (*N* = 33), with or without mutations in the ATP-sensitive K^+^ channel genes, *ABCC8*, and *KCNJ11* requiring glucagon by dose titration in addition to intravenous dextrose and medical therapy with diazoxide/octreotide to achieve glycemic stability were recruited. Following glucagon titration and a 24-h glucose stable period, glucose infusion rate (GIR) was reduced over a 24-h period. Achievement of glycemic stability and decrease in GIR were considered end points of the study.

**Results:** All patients achieved glycemic stability with glucagon infusion, demonstrating clinical benefit. GIR reduced from 15.6 (4.5) to 13.4 (4.6) mg/kg/min mean (SD) (*p* = 0.00019 for difference; *n* = 32; paired *t*-test) over 24 h. By univariate analysis, no individual baseline characteristic was associated with changes in the GIR. However, by baseline-adjusted modeling, mutational status of the patient (*p* = 0.011) was inversely associated with a reduction in GIR. Adverse events were infrequent with diarrhea possibly attributed to glucagon treatment in 1 patient. With long-term treatment following GIR reduction, necrolytic migratory erythema was observed in another patient.

**Conclusion:** These data suggest that dose-titrated glucagon infusion therapy aids hypoglycemia prevention and reduction in GIR in the clinical management of patients with CHI.

## Background

Congenital hyperinsulinism (CHI) is a rare disease, with an annual incidence of ~1/30,000 live births in non-consanguineous populations (1); however, it represents the most-common cause of severe and recurrent hypoglycemia in infants and children (2). CHI is caused by dysregulated and excess secretion of insulin from pancreatic β-cells in patients with or without genetic mutations in ATP-sensitive K^+^ channels (3). This uncontrolled release of insulin in CHI can lead to frequent and severe bouts of hypoglycemia, which in turn can have severe and lasting consequences on central nervous system (CNS) function and neurodevelopment in young children (4). These consequences include elevated risks for seizures, delayed developmental milestones, and ongoing CNS injury (4–6). It is important to note that these adverse impacts on CNS function and development have been reported in patients with either transient or permanent forms of CHI (4), and in patients with either focal or diffuse CHI (5). In light of the gravity and lifelong impact of the adverse effects on early CNS development resulting from severe hypoglycemia in infants, prevention, and risk mitigation are critically important for the preservation of CNS function in affected children (2, 6).

CHI is often treated with dextrose infusions to rapidly correct hypoglycemia, and to also maintain normal plasma glucose concentrations. However, while this approach is a mainstay of the clinical management of children with CHI, it is limited in part by the total volume that can be administered to small children (2, 7). To help address the above concerns regarding the long-term consequences of repeated, severe bouts of hypoglycemia, glucagon is widely used “off-label” for the early management of ongoing hypoglycemia in CHI. As a counter-regulatory hormone to insulin, glucagon stimulates glycogenolysis and gluconeogenesis, while also inhibiting glycogenesis (8, 9).

Glucagon therapy prevents hypoglycemia during a critical period of neurological development by reversing the blunted counter-regulatory response so common in CHI (10). Therefore, glucagon has the potential to prevent and reduce neuroglycopenia-induced, long-range neurodevelopmental outcomes (10). However, to date, there are a limited number of reports describing the efficacy of glucagon therapy in this setting.

In a recent study, glucagon infusion at a fixed rate led to the reduction of the glucose infusion rate (GIR) within 24 h of initiating treatment in patients with CHI (7). However, this study did not investigate the efficacy of glucagon dose-titrating regimens, commonly used in many centers. Here we report the efficacy of glucagon in a dose-titrating regimen to demonstrate real-world therapeutic benefit that may support design and clinical development of novel glucagon analogs in patients with CHI: [https://clinicaltrials.gov/ct2/show/NCT03777176].

The objective of this case series review was to assess the potential clinical utility of dose-titrated glucagon infusion regimens to achieve glycemic stability, and prevent hypoglycemia in patients with CHI.

## Methods

### Patients

CHI was diagnosed according to standard criteria (i.e., the identification of detectable serum insulin concentrations during a hypoglycemic episode, along with high glucose requirement, hypoketosis, and recurrence of hypoglycemia in the absence of other causes of hypoglycemia) (2) in infants and children from local hospitals and neonatal units. All patients who required glucagon therapy for glycemic stabilization, in addition to intravenous (IV) dextrose infusions and medical therapy (diazoxide and/or octreotide) were eligible for recruitment to the study. Patients suspected to have genetic syndromes with prior risk of hypoglycemia were excluded from the study. Patients likely to have mild transient CHI (e.g., infants of diabetic mothers, CHI due to perinatal stress) were also excluded from the study. Birth weight was not a criteria for recruitment; small for gestational age children who had severe hypoglycemia and therefore transferred to the service for clinical management were included in the study.

Patient demographics and baseline characteristics including patient age, sex, weight, relevant diagnoses (CHI, focal/diffuse, transient, or persistent, etc.) were collected from existing patients' medical records according to standard clinical practice.

Following informed consent, patients awaiting pancreatic surgery (either focal lesionectomy or subtotal pancreatectomy), as well as patients with severe hypoglycemia not requiring pancreatic surgery who were receiving a continuous infusion of glucagon were recruited between 2007 and 2015. For each patient assigned to a surgical intervention, 2 patients undergoing medical therapy were also recruited.

For the purposes of this study, a patient was considered to have transient CHI if their hypoglycemia resolved, and they achieved glycemic stability with no ongoing requirement for medical or surgical treatment. In the absence of a consensus definition, transient CHI was not time limited but excluded those in whom hyperinsulinism resolved with no dependence on medication within 2 weeks of diagnosis. In contrast, persistent CHI was considered if patients underwent either surgical treatment or continued to receive medical therapy at the time of writing the manuscript. Therefore, the diagnoses of either transient or persistent CHI were made in retrospect, and not at the time of glucagon therapy.

### Mutation Status

Patient DNA samples were analyzed for mutations by Sanger sequencing of *ABCC8* and *KCNJ11* at the University of Exeter Molecular Genetics Laboratory. In patients without *ABCC8/KCNJ11* mutations, targeted gene panel testing for mutations associated with CHI was undertaken.

### Study Design and Protocol

**Figure 1:**
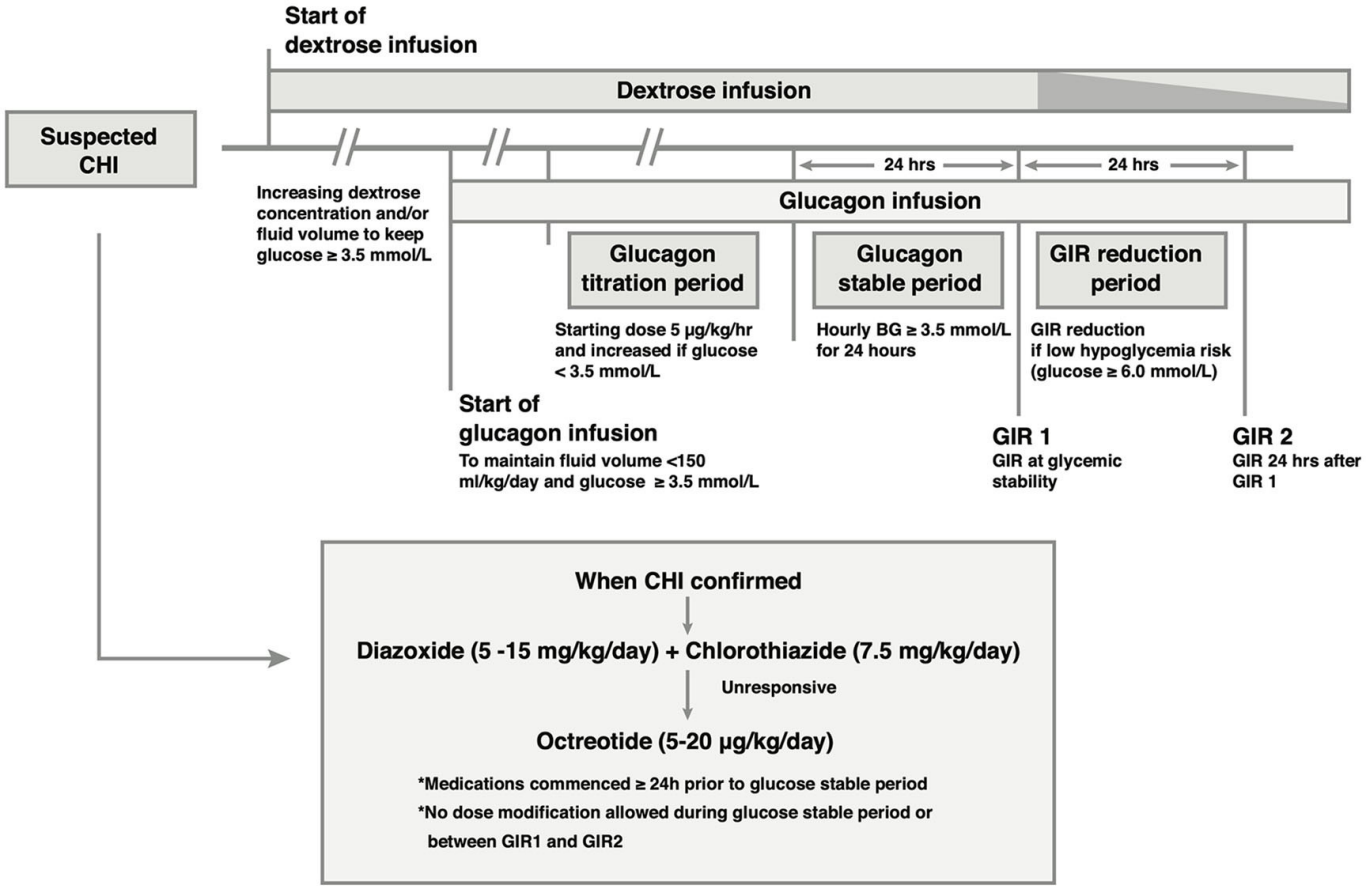
Outline of the medical treatment and glucagon infusion protocol. Glucagon is added to the intravenous (IV) drug regimen if glycemic stability is not achieved with IV dextrose. Once the diagnosis of CHI is established, CHI-specific medication is commenced, and titrated to glycemic response independent of glucagon dose in the period leading up to the glucose stable period. In the glucose stable period, medication dose is unaltered. Following achievement of glucose stability, IV dextrose may be reduced if glucose concentration is ≥ 6.0 mmol/L, resulting in a reduction in the Glucose Infusion Rate (GIR) (speckled taper) without reducing medication doses.

This clinical case series was designed as a prospective observational study. Some data were collected retrospectively to allow the attending physician to modify any clinical variables under investigation based on the medical needs of the patient. The general outline of the clinical approach to severe hypoglycemia and glucagon therapy protocols for the study are shown in Figure 1, Supplementary Figure 1.

#### Dextrose Infusion

The standard of care for patients diagnosed with suspected CHI was to initiate a 10% dextrose infusion via a peripheral/central venous cannula at a standard maintenance rate (usually 60 ml/kg/day on Day 1 of life in term neonates). If hypoglycemia was not corrected, then fluid volumes and/or dextrose concentrations were escalated as needed to achieve glycemic stability (plasma glucose ≥ 3.5 mmol/L).

#### Concomitant Medications

- **Diazoxide:** Following their diagnosis of CHI, all patients were initiated on oral diazoxide therapy (specified as Proglycem®, Teva Pharmaceuticals, USA). Patients were commenced at a dose of 5 mg/kg/day with additional echocardiography monitoring for evaluation of cardiac status, anatomical congenital heart defects, and risk of pulmonary hypertension. The diazoxide dose was only escalated at the discretion of the clinician and was not standardized or incorporated into the glucagon dose protocol. Importantly, the dose of diazoxide was left unchanged during the glucose stable period and the GIR reduction period (Figure 1). The maximum dose of diazoxide was recorded for purposes of the study; the timing of the maximum dose was irrespective of the timing of GIR2. Chlorothiazide was added to diazoxide therapy for diuretic action and synergistic effect at a dose of 7.5 mg/kg/day that was not altered during glucagon therapy.
- **Octreotide:** Therapy with octreotide was initiated following establishment of diazoxide unresponsiveness. The starting dose of octreotide was 5 μg/kg/day in 4 divided subcutaneous bolus doses.

The principle of clinical management was to achieve glycemic stability and prevent hypoglycemia in the shortest possible time to minimize the long-term risk of neuroglycopenia. The protocol allowed for clinician-dependent decisions for dextrose infusions and diazoxide/octreotide dosing in the glucagon titration period leading up to the glucose stable period. Following the achievement of glucose stability, GIR reduction was allowed if the clinician (pediatric endocrinologist) perceived a low risk of hypoglycemia. A low risk was deemed for plasma glucose concentrations ≥ 6 mmol/L.

#### Intervention: Glucagon Infusion Protocol

Once patients were established on a dextrose infusion, and irrespective of diazoxide efficacy, they were initiated on a titrated dose regimen of glucagon (beginning at 5 μg/kg/h, and escalated as needed). The 2 main goals were to (1) achieve normal plasma glucose concentrations as quickly as possible, and (2) reduce large fluid infusion volumes (Figure 1).

The dose of glucagon was drawn up and diluted with 0.9% sodium chloride solution to make up to 5 ml in a 10 ml syringe to give an initial rate of 0.2 ml/h equating to 5 μg/kg/h. Glucagon solutions were changed every 12 h due to glucagon instability in prepared solutions. Glucagon was delivered via peripheral intravenous (IV) infusion in the majority, with hourly plasma glucose monitoring by uniform point of care testing (Nova Biomedical Inc). The titration strategy was to achieve plasma glucose concentrations ≥3.5 mmol/L while ensuring that the total fluid volume remained ≤150 mL/kg/day to prevent fluid overload. If glucose concentrations fell below 3.5 mmol/L, an IV bolus of 10% dextrose (2 mL/kg) was administered. The dose of glucagon was reviewed every 4 h.

In cases where a supplemental glucose bolus was required, glucagon was dose escalated in steps of 2.5 μg/kg/h until stable glucose concentrations were achieved in the period prior to the glucose stable period. Once glycemic stability was achieved for 4 h, the glucose stable period was extended to 24 h. The maximum dose of glucagon allowed was 30 μg/kg/h.

During the glucagon titration period leading up to the glucose stable period, titration of dextrose/diazoxide/octreotide was allowed as per clinician's preference and was not stipulated in the glucagon protocol. Dextrose and diazoxide were used for at least 24 h prior to the glucose stable period.

The study did not incorporate a control arm as it was not feasible to test glycemic stability without glucagon. As glucagon is standard therapy in patients with CHI (although efficacy has not been reported in titration dosing) it was deemed impossible to deprive patients of therapy that aims to normalize glucose concentrations as rapidly as possible in order to prevent neuroglycopenia and brain injury. Instead, a case series of patients were utilized in an observational design that would reflect real-world scenarios in a rare and complex disease with lifelong and life-threatening consequences.

### Study Endpoints

The rate of dextrose infusion in ml/kg/day was recorded as the GIR. GIR was comprised of the total dextrose administered, including IV dextrose, parenteral nutrition and enteral intake where applicable. Glycaemic stability was defined as hourly serial plasma glucose concentrations ≥ 3.5 mmol/L for 24 h. The GIR at the end of this 24-h period was defined as GIR1.

Reduction of IV GIR (keeping enteral GIR stable) was attempted in the following 24-h period, and this was recorded as GIR2. GIR reduction was at the clinician's discretion; the degree and/or rate of GIR reductions were not stipulated by center guidelines but a reduction in GIR was suggested if risk of hypoglycemia was deemed to be low (plasma glucose ≥ 6 mmol/L).

The highest dose of glucagon (μg/kg/hour) required to prevent hypoglycemia (defined as plasma glucose < 3.5 mmol/L, measured by 1-h point-of-care testing) during the glucagon titration period was recorded. The goal for this treatment regimen was to keep the patient's plasma glucose concentrations ≥ 3.5 mmol/L. The first end point of the study was to demonstrate glycemic stability by dose-titrated glucagon and the second end point was to demonstrate a reduction in GIR. Additionally, AEs were recorded; including the presence of convulsions (as a consequence of hypoglycemic episodes), and the presence of diarrhea and/or rash.

### Statistical Analysis

Differences in GIR between the 2 timepoints (GIR1 and GIR2; change in GIR) were investigated using a paired *t*-test.

Associations between differences in GIR and baseline variables were investigated using multiple regression models with change in GIR as the dependent variable and using different combinations of covariates. Individual comparisons of 2 categorical variables were assessed using the Fisher's exact test.

All analyses were performed using the R statistical package (version 3.6.0 for Microsoft Windows 64-bit). A *p* < 0.05 was considered to indicate statistical significance.

Data are reported as mean (SD), except where median (range) values are more useful to appreciate the range of skewed distributions.

## Results

### Patients

A total of 33 patients were recruited to this study, of which 24 (72.7%) were male (Table 1). Median (range) birthweight was 3.3 kg (1.6–5.4). Patients presented with hypoglycemia at a median (range) age of 1.0 (1.0–366.0) days. For this group of patients, the majority presented during the neonatal period, while 3 patients presented in late infancy. At initial presentation to the hospital, mean (SD) patient glucose concentrations were 1.4 (0.8) mmol/L, while corresponding insulin concentrations were 17.8 (15.9) pmol/L. The proportions of patients with transient vs. persistent CHI were evenly distributed in this cohort, with 16 (48.5%) diagnosed with transient and 17 (51.5%) with persistent disease. Convulsions were an infrequent occurrence, being reported in only 6 (18.2%) patients.

**Table 1:** Patient baseline characteristics.

	**Overall (*n* = 33)**
**Gender**	
Male	24 (72.7%)
Female	9 (27.3%)
**Birthweight (kg)**	
Mean (SD)	3.29 (0.93)
Median [Min, Max]	3.34 [1.57, 5.40]
Missing	2 (6.1%)
**Age at diagnosis (days)**	
Mean (SD)	22.8 (74.3)
Median [Min, Max]	1 [1, 366]
**Convulsions**	
No	27 (81.8%)
Yes	6 (18.2%)
**Mutation status**	
No mutation	23 (69.7%)
Any mutation	10 (30.3%)
**Glucose at diagnosis (mmol/L)**	
Mean (SD)	1.39 (0.82)
Median [Min, Max]	1.35 [0.10, 4.50]
Missing	1 (3.0%)
**Insulin concentration at diagnosis (pmol/L)**	
Mean (SD)	17.8 (15.9)
Median [Min, Max]	12.8 [2.1, 61.1]
Missing	4 (12.1%)
**Glucagon dose at stabilization (μg/kg/h)**	
Mean (SD)	12.4 (5.9)
Median [Min, Max]	12.0 [5.0, 20.0]
**Duration of glucagon use (days)**	
Mean (SD)	33.5 (50.1)
Median [Min, Max]	11 [2, 205]
Missing	14 (42.4%)
**Diazoxide dose (mg/kg/day)**	
Mean (SD)	8.97 (4.54)
Median [Min, Max]	7.00 [4.00, 15.00]
**Any Octreotide dose**	
No	22 (66.7%)
Yes	11 (33.3%)

### Mutation Status

Since mutation status was not available at the time of treatment, all therapeutic approaches employed in this study were “mutation status blind.” Subsequent to the conduct of the clinical treatment of the patients, a genetic cause of CHI due to mutations in the ATP-sensitive K^+^ channel subunits (*KCNJ11* and *ABCC8*) was identified in 10 patients. Of the 6 patients with monoallelic mutations, 5 had focal CHI and underwent focal lesionectomy.

### Treatments

Glucagon was administered as an IV infusion in 31 patients, while 2 patients received their glucagon infusion subcutaneously. Glucagon was infused for a median (range) duration of 11 (2–205) days. With the addition of titrated glucagon, hypoglycemia was arrested in all patients, who demonstrated plasma glucose concentrations ranging between 3.5 and 10.0 mmol/L. The titrated dose of glucagon at the start of the glucose stable period was 12.4 (5.8) μg/kg/h. Glucagon infusions allowed for fluid restriction with total fluid intake not exceeding 150 ml/kg/day in all patients. No patient in the study developed features of heart failure or pulmonary hypertension further adding to the efficacy of glucagon in maintaining stable glycaemia without excessive volume intake.

**Figure 2:**
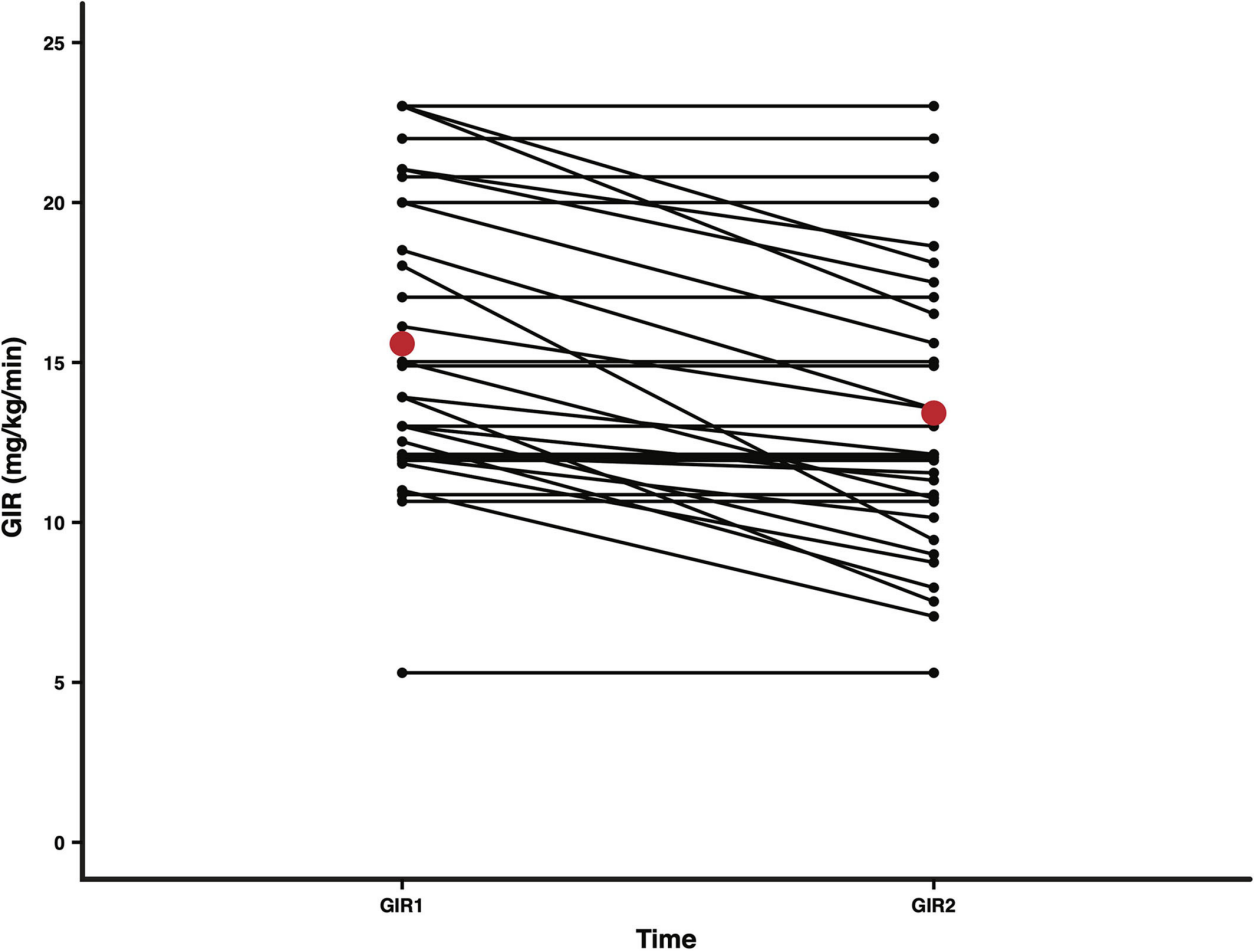
Mean GIRs at two time points, 24 h apart, after achieving glycemic stability in all patients. A reduction in IV dextrose was attempted at the end of the glucose stable period to reduce GIR (mg/kg/min). GIR1 and GIR2 representing GIR values for each patient at the start and end of the GIR reduction period (*n* = 32; 1 patient did not have paired GIR1 and GIR2 values) are presented as black dots. Mean GIR1 (15.6) and GIR2 (13.4) values are presented as red dots. The mean difference in GIR over the 24 h was 2.2 (*p* = 0.000019; paired *t*-test).

Glycaemic stabilization by glucagon titration led to a reduction in the GIR from 15.6 (4.5) to 13.4 (4.5) mg/kg/min (*p* = 0.000019 for difference; paired *t*-test) within 24 h in the 32 patients for whom paired GIR1 and GIR2 values were available (Figure 2). GIR reduced in 18 (56.2%) patients, but no change in the GIR was reported in 14 (43.8%) patients.

GIR reduced in 50% of those requiring surgical treatment indicating that the effect was directly attributable to glucagon and therefore unlikely due to a reduction in severity of hyperinsulinism. Further, severity of hypoglycemia was similar between those with transient and persistent CHI with no difference in GIR (mean GIR 15.7 vs. 15.4, *p* = 0.73), indicating that patients with transient CHI were not already on a reducing GIR trajectory.

Pancreatic surgery was required in 11 patients. In 6 patients with diffuse or atypical CHI subtotal pancreatectomy was performed. In 5 patients with focal CHI, focal lesionectomy was successfully performed to treat hypoglycemia. In the rest of the patients, medical therapy was effective in achieving glycemic stability. In 22 patients managed by medical therapy, CHI resolved in 16 after a mean (SD) 52.7 (89.5) weeks, without the need for ongoing requirement for regular medications.

### Correlates to GIR Reduction

**Figure 3:**
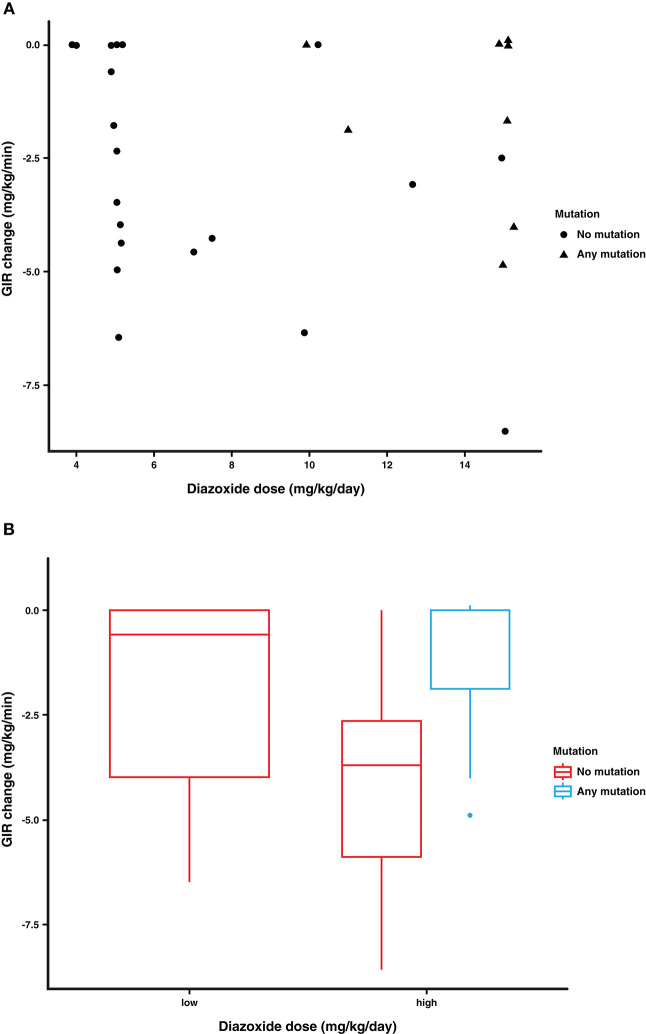
Association of diazoxide dose and gene mutation with change in GIR in response to glucagon treatment. The change in GIR (mg/kg/min) (GIR1- GIR2) was analyzed in relation to the patient's diazoxide dose (highest dose received), and their mutation status (*ABCC8, KCNJ11*). **(A)** Patients with gene mutations in their ATP-sensitive K^+^ channels received higher doses of diazoxide and demonstrated less robust changes in their GIR. **(B)** Low and high diazoxide doses are based on a cut off dose of 7.5 mg/kg/day and reveal that even patients with *ABCC8/KCNJ11* mutations are glucagon responsive.

Univariate regression modeling did not identify patient baseline characteristics or therapeutic modalities that were associated with patient outcomes. However, when a baseline-adjusted modeling approach (GIR change = GIR1 + mutation + diazoxide dose) was tested, changes in the patients' GIR (GIR1- GIR2), in relation to the highest dose of diazoxide received and the presence or absence of mutations in their ATP-sensitive K^+^ channels, significant associations were identified (mutation *p* = 0.011, diazoxide dose *p* = 0.022). As expected, patients with mutations in their ATP-sensitive K^+^ channels received higher doses of diazoxide in the hospital and demonstrated less robust reductions in GIR (Figure 3).

### Concomitant Medications

The maximum diazoxide dose was 8.9 (4.5) mg/kg/day, with clinician-optimized dose titrations in the period leading up the glucose stable period (Table 1). Eleven patients were unresponsive to diazoxide and required second-line treatment with octreotide, up to a maximum dose of 20.0 μg/kg/day. Patients recruited to the study did not demonstrate AEs to preclude or stop diazoxide therapy. Octreotide therapy in those non-responsive to diazoxide (*n* = 11, any dose) was correlated with mutation status by Fisher's exact test (two-tailed; *p* = 0.0004), indicating its correlation with greater severity of CHI.

### AEs

AEs associated with glucagon treatment were infrequent; 1 child had diarrhea, possibly related to glucagon treatment. Another child exhibited necrolytic migratory erythema while receiving glucagon over a period of 60 days. The maximum dose for this patient was 20 μg/kg/h. The rash disappeared once glucagon therapy was stopped. In this patient cohort, no cases of thrombocytopaenia secondary to glucagon treatment were reported.

## Discussion

Our study demonstrates the efficacy of continuous IV glucagon in the management of hypoglycemia due to CHI. In our case series, titrated glucagon was successful as an adjunct in the stabilization of glucose concentrations and prevention of hypoglycemia while reducing dependence of high volumes of dextrose containing fluid. Using a glucagon protocol that allowed dextrose and medication optimization preceding a 24-h glucose stability period followed by a 24-h GIR reduction period, all patients ceased to have hypoglycemia, indicating efficacy of glucagon. In addition, glucagon enabled the reduction in GIR in more than half of patients, including those in whom pancreatic surgery was subsequently required.

The present study confirms previous observations regarding the efficacy of glucagon in achieving glycemic stability, and in reducing GIRs, while potentially extending the efficacy to wider clinical applications. Dose titration of glucagon infusions is commonly performed in the management of patients with CHI and is clinically relevant, but this approach has not been previously reported.

A recent report focused on the treatment of a cohort of patients awaiting pancreatic surgery with glucagon at a standard dose without titration, recording reductions in the GIR over 24 h (6). Therefore, our study supports the validity of titration of glucagon in the treatment of hypoglycemia alongside the use of glucagon at a standardized dose. Our observational study also supports the application of titrated glucagon infusion in patients with a wide spectrum of CHI, i.e., in patients who were both responsive and unresponsive to standard medical therapy, patients with and without genetic mutations and those with transient or persistent forms of CHI. Our data reflects real world clinical scenarios where patients are initiated on multiple therapies simultaneously with the sole objective to normalize glucose concentration as rapidly as possible.

In our study, we analyzed patient factors that might be associated with the clinical severity of CHI, including standard patient demographics and baseline characteristics, the presence of genetic mutations in the *ABCC8*/*KCNJ11* genes, and the use of concomitant medications such as diazoxide and/or octreotide. By direct analysis, none of these characteristics were individually associated with changes in the GIR in response to glucagon treatment. However, by baseline-adjusted modeling, ATP-sensitive K^+^ channel mutations were associated with a limited reduction in the GIR in response to glucagon infusion, as expected. An important implication of these data is that mutations in the ATP-sensitive K^+^ channel, a surrogate marker of CHI severity, are associated with limited reductions in the GIR in response to glucagon, indicating their potential predictive value for therapeutic responses to glucagon treatment.

We acknowledge that our observational study has limitations, including the absence of controls and the relatively modest number of patients in the dataset. The absence of controls does not completely negate the possibility that reduction in GIR may be attributed to reduction in disease severity or due to a delayed diazoxide effect. However, it is difficult to justify a control arm for standard of care medication (even though efficacy of dose titration of glucagon remains unproven) in a disease where medications are simultaneously used in order to reduce the risk of hypoglycemia in the shortest possible time. For the same reason, it was not possible to stipulate a uniform dose for diazoxide and/or octreotide. Even without a control arm in our observational study design, it was clear that glucagon effect was likely.

The magnitude of the reduction in GIR was modest (14%) but this was likely to be a consequence of cautious reduction by clinicians who aimed to minimize hypoglycemia risk. Further the duration of reduction was limited to 24 h; a longer duration might have demonstrated a larger magnitude of reduction. Nonetheless, reduction in GIR occurred both in transient and persistent CHI and occurred even in those with mutations and those requiring pancreatic surgery. Further, GIR prior to dose reduction was similar in those with transient and persistent CHI, implying that the trajectory of disease variation was no different between groups at the start of the GIR reduction period, adding evidence to an observed glucagon effect.

It is possible that GIR reduction was correlated to a diazoxide effect rather than being a glucagon effect. However, in all cases diazoxide was commenced at least 24 h prior to the glucose stable period. Therefore, by the start of the GIR reduction period, patients would have received at least 48 h of diazoxide therapy with no dose escalation in the previous 24 h to significantly impact GIR reduction. Further, GIR reduction was also observed even in those unresponsive to diazoxide, implying a valid glucagon effect independent of diazoxide efficacy.

While glucagon was found to be effective and reduced hypoglycemia risk in all patients, we observed 1 episode of necrolytic migratory erythema in a patient on high-dose prolonged glucagon infusion therapy. While the incidence of AEs from glucagon use was infrequent (1 other child had diarrhea, while none had thrombocytopenia), it is important to be aware of the possibility of AEs from longer-term use of glucagon infusion therapy.

Our study supports earlier findings where fixed dose glucagon was successfully used to reduce the rate of hypoglycemic events in patients with CHI (7). It remains to be seen if similar responses to glucagon, and titrated glucagon infusions in particular, will be observed in other cohorts. It will be especially interesting to review patient responses to novel glucagon analogs presently in clinical development (11).

We conclude that dose-titrated glucagon infusion therapy can be clinically effective for glycemic stabilization in patients with CHI. The use of glucagon is generally safe, but the limited solubility in aqueous solutions, and the possibility of AEs, including necrolytic migratory erythema, need consideration.

## Data Availability Statement

The raw data supporting the conclusions of this article will be made available by the authors, without undue reservation.

## Ethics Statement

This study was reviewed by the North West Research Ethics Committee (project reference number: 07/H1010/88) to which parents were consented to participate.

## Author Contributions

MS-E, IB, and MD conceived the study. IB and MS-E was responsible for the design of the study. DY collected data and assisted in writing the manuscript. SB conducted the statistical analysis. MS-E, MD, JW, and IB wrote and corrected the manuscript. All authors contributed to the article and approved the submitted version.

## Conflict of Interest

SB is employed by Zealand Pharma A/S of Søborg, Denmark, which is involved in the development of a glucagon analog. JW is employed by rareLife solutions, a medical writing service based in S. Norwalk, CT, USA. The remaining authors declare that the research was conducted in the absence of any commercial or financial relationships that could be construed as a potential conflict of interest.

## Supplementary Material

Supplemental Figure 1 Representation of the initial management of patients presenting with severe hypoglycemia in our institution. Dextrose infusion is commenced followed by glucagon infusion in order to maintain glucose concentration ≥ 3.5 mmol/L. When hyperinsulinism is confirmed, CHI-specific medication is initiated and then adjusted according to clinical response.

## Abbreviations

AE: adverse event
CHI: congenital hyperinsulinism
CNS: central nervous system
GIR: glucose infusion rate
SD: standard deviation.
