# Corrigendum: Efficacy of Dose-Titrated Glucagon Infusions in the Management of Congenital Hyperinsulinism: A Case Series

**DOI:** 10.3389/fendo.2020.614734

**Published:** 2020-11-16

**Authors:** Maria Salomon-Estebanez, Daphne Yau, Mark J. Dunne, Chris Worth, Sune Birch, José L. Walewski, Indraneel Banerjee

**Affiliations:** ^1^ Department of Paediatric Endocrinology, Royal Manchester Children’s Hospital, Manchester, United Kingdom; ^2^ Faculty of Biology, Medicine and Health, University of Manchester, Manchester, United Kingdom; ^3^ Department of Statistics, Zealand Pharma A/S, Søborg, Denmark; ^4^ Medical Publications, rareLife solutions, Norwalk, CT, United States

**Keywords:** glucose, congenital hyperinsulinism, hypoglycemia, glucagon infusion, dose titration

In the original article, there was a mistake in **Figure 1** and its caption as published.

**Figure 1:**
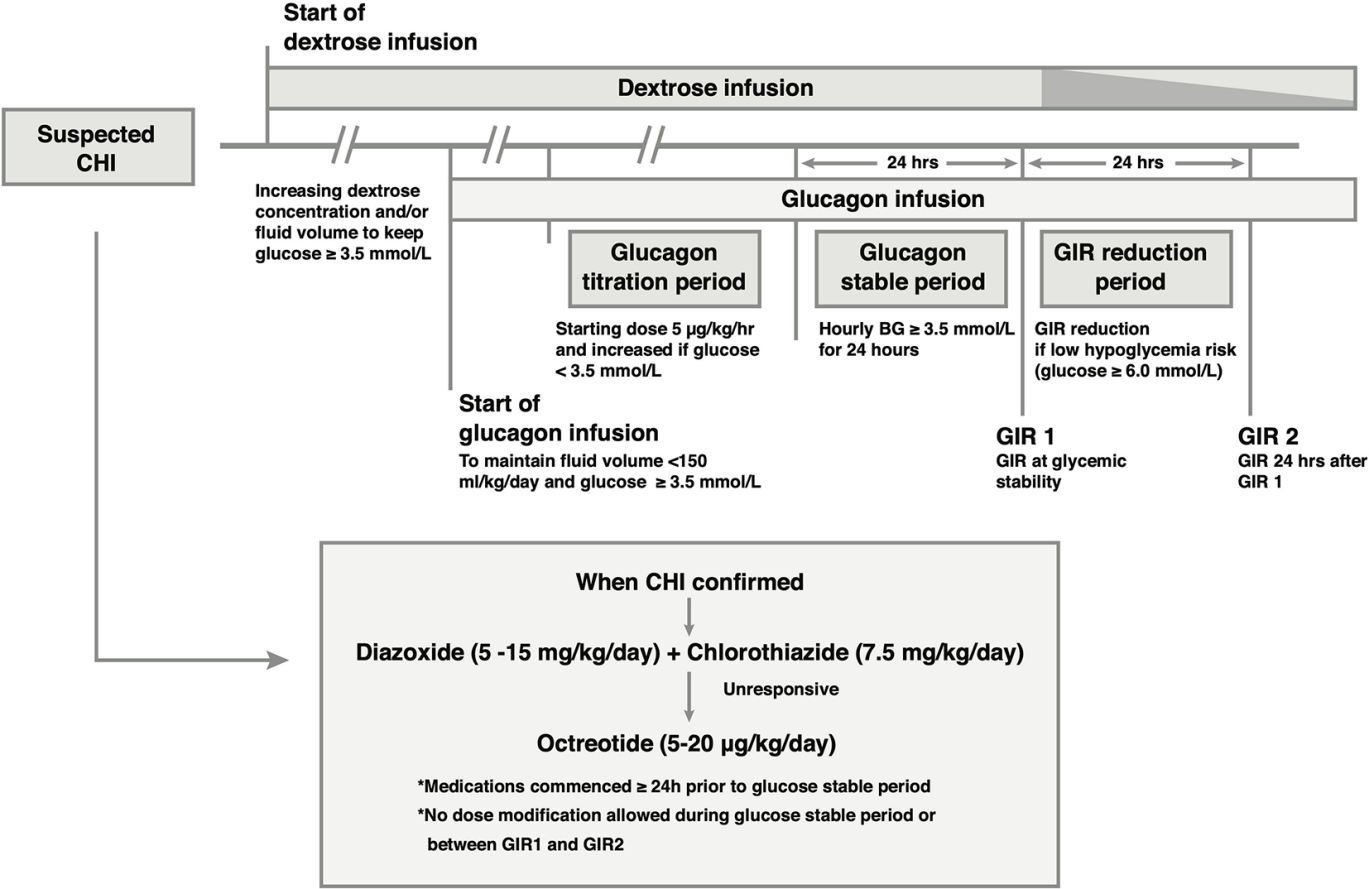
Outline of the medical treatment and glucagon infusion protocol. Glucagon is added to the intravenous (IV) drug regimen if glycemic stability is not achieved with IV dextrose. Once the diagnosis of CHI is established, CHI-specific medication is commenced, and titrated to glycemic response independent of glucagon dose in the period leading up to the glucose stable period. In the glucose stable period, medication dose is unaltered. Following achievement of glucose stability, IV dextrose may be reduced if glucose concentration is ≥ 6.0 mmol/L, resulting in a reduction in the Glucose Infusion Rate (GIR) (speckled taper) without reducing medication doses.

In the figure, the “>” symbols should be “≥”.

In the legend, the last sentence should read “…if glucose concentration is ≥ 6.0 mmol/L, …”.

The correct **Figure 1** and legend appear below.

In the original article, there was a mistake in **Figure 2** and its caption as published.

**Figure 2:**
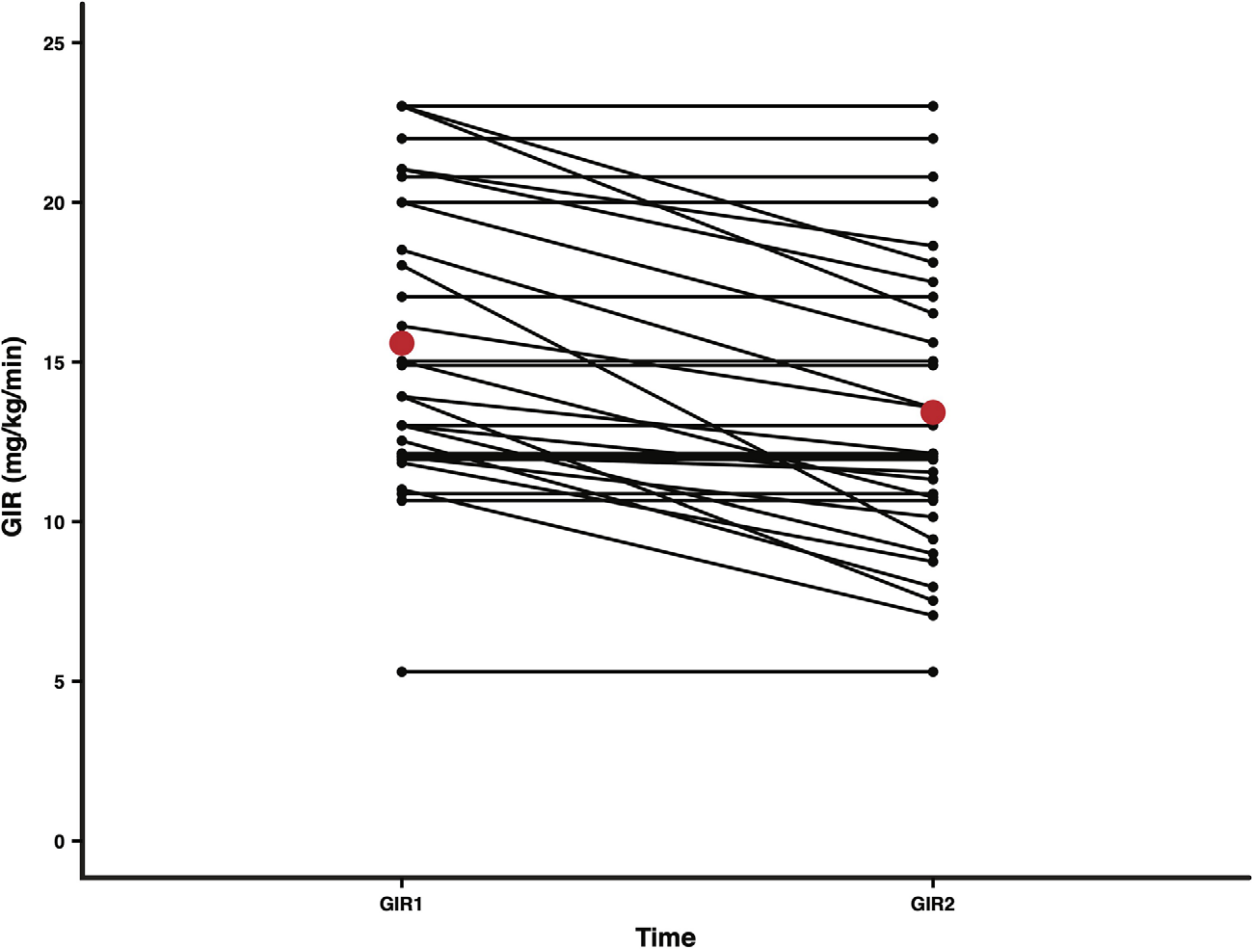
Mean GIRs at two time points, 24 h apart, after achieving glycemic stability in all patients. A reduction in IV dextrose was attempted at the end of the glucose stable period to reduce GIR (mg/kg/min). GIR1 and GIR2 representing GIR values for each patient at the start and end of the GIR reduction period (*n* = 32; 1 patient did not have paired GIR1 and GIR2 values) are presented as black dots. Mean GIR1 (15.6) and GIR2 (13.4) values are presented as red dots. The mean difference in GIR over the 24 h was 2.2 (*p* = 0.000019; paired *t*-test).

In the figure, the Y-axis label incorrectly included “change”. Also, the X-Axis had a typo; the righthand tic mark should be GIR2 instead of GIR1.

In the legend “Mean Change in GIR after achieving glycemic stability (all patients)” was incorrect and should have read “Mean GIRs at two time points, 24 h apart, after achieving glycemic stability in all patients”.

The correct **Figure 2** and legend appear below.

In the original article, there was a mistake in the **Supplementary Figure 1** and its caption as published.

In the figure, the criteria for Dextrose Infusion, and Glucagon Infusion have incorrect “>” symbols before 3.5 mmol/L and should be “≥”.

In the legend, the same error occurred.

The correct **Supplementary Figure 1** and legend appear below.

**Supplementary Figure 1:**
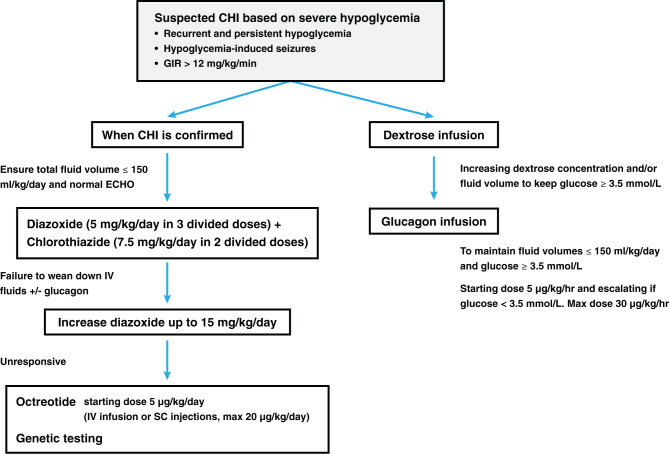
Representation of the initial management of patients presenting with severe hypoglycemia in our institution. Dextrose infusion is commenced followed by glucagon infusion in order to maintain glucose concentration ≥ 3.5 mmol/L. When hyperinsulinism is confirmed, CHI-specific medication is initiated and then adjusted according to clinical response.

## Text Corrections

In the original article, a few typos were noted.

In **Methods, Concomitant Medications,** **Paragraph 3**, the incorrect symbol “>” should have read “≥”. The correct text appears below:

The principle of clinical management was to achieve glycemic stability and prevent hypoglycemia in the shortest possible time to minimize the long-term risk of neuroglycopenia. The protocol allowed for clinician-dependent decisions for dextrose infusions and diazoxide/octreotide dosing in the glucagon titration period leading up to the glucose stable period. Following the achievement of glucose stability, GIR reduction was allowed if the clinician (pediatric endocrinologist) perceived a low risk of hypoglycemia. A low risk was deemed for plasma glucose concentrations ≥ 6 mmol/L.

In **Methods, Intervention: Glucagon Infusion Protocol, Paragraph 2**, the incorrect symbol “>” should have read “≥”. The correct text appears below:

The dose of glucagon was drawn up and diluted with 0.9% sodium chloride solution to make up to 5ml in a 10ml syringe to give an initial rate of 0.2 ml/h equating to 5 μg/kg/h. Glucagon solutions were changed every 12 h due to glucagon instability in prepared solutions. Glucagon was delivered *via* peripheral intravenous (IV) infusion in the majority, with hourly plasma glucose monitoring by uniform point of care testing (Nova Biomedical Inc). The titration strategy was to achieve plasma glucose concentrations ≥ 3.5 mmol/L while ensuring that the total fluid volume remained ≤ 150 mL/kg/day to prevent fluid overload. If glucose concentrations fell below 3.5 mmol/L, an IV bolus of 10% dextrose (2 mL/kg) was administered. The dose of glucagon was reviewed every 4 h.

In **Methods, Study endpoints, Paragraph 1**, the incorrect term “compromised” should have read “comprised”. The correct text appears below:

The rate of dextrose infusion in ml/kg/day was recorded as the GIR. GIR was comprised of the total dextrose administered, including IV dextrose, parenteral nutrition and enteral intake where applicable. Glycaemic stability was defined as hourly serial plasma glucose concentrations ≥ 3.5 mmol/L for 24 h. The GIR at the end of this 24-h period was defined as GIR1.**

In **Methods, Study endpoints, Paragraph 2**, the incorrect symbol “>” should have read “≥”. The correct text appears below:

Reduction of IV GIR (keeping enteral GIR stable) was attempted in the following 24-h period, and this was recorded as GIR2. GIR reduction was at the clinician’s discretion; the degree and/or rate of GIR reductions were not stipulated by center guidelines but a reduction in GIR was suggested if risk of hypoglycemia was deemed to be low (plasma glucose ≥ 6 mmol/L).

In **Methods, Study endpoints, Paragraph 3**, the incorrect symbol “>” should have read “≥”. The correct text appears below:

The highest dose of glucagon (μg/kg/hour) required to prevent hypoglycemia (defined as plasma glucose < 3.5 mmol/L, measured by 1-h point-of-care testing) during the glucagon titration period was recorded. The goal for this treatment regimen was to keep the patient’s plasma glucose concentrations ≥ 3.5 mmol/L. The first end point of the study was to demonstrate glycemic stability by dose-titrated glucagon and the second end point was to demonstrate a reduction in GIR.

In **Results, Mutation Status, Paragraph 1**, the terms “to the conduct of” were missing. The correct text appears below:

Since mutation status was not available at the time of treatment, all therapeutic approaches employed in this study were “mutation status blind.” Subsequent to the conduct of the clinical treatment of the patients, a genetic cause of CHI due to mutations in the ATP-sensitive K+ channel subunits (KCNJ11 and ABCC8) was identified in 10 patients.Of the 6 patients with monoallelic mutations, 5 had focal CHI and underwent focal lesionectomy.

In **Results, Treatments, Paragraph 2**, the incorrect value “15.5” should have read “15.6”. The correct text appears below:

Glycaemic stabilization by glucagon titration led to a reduction in the GIR from 15.6 (4.5) to 13.4 (4.5) mg/kg/min (p = 0.000019 for difference; paired t-test) within 24 h in the 32 patients for whom paired GIR1 and GIR2 values were available (**Figure 2**). GIR reduced in 18 (56.2%) patients, but no change in the GIR was reported in 14 (43.8%) patients.

The authors apologize for these errors and state that they do not change the scientific conclusions of the article in any way. The original article has been updated.

