# Supplementary figures and images for: Efficacy of Dose-Titrated Glucagon Infusions in the Management of Congenital Hyperinsulinism: A Case Series
Source: Front Endocrinol (Lausanne). 2020 Sep 3;11:441. doi: 10.3389/fendo.2020.00441 (PMC7494759; doi:10.3389/fendo.2020.00441)

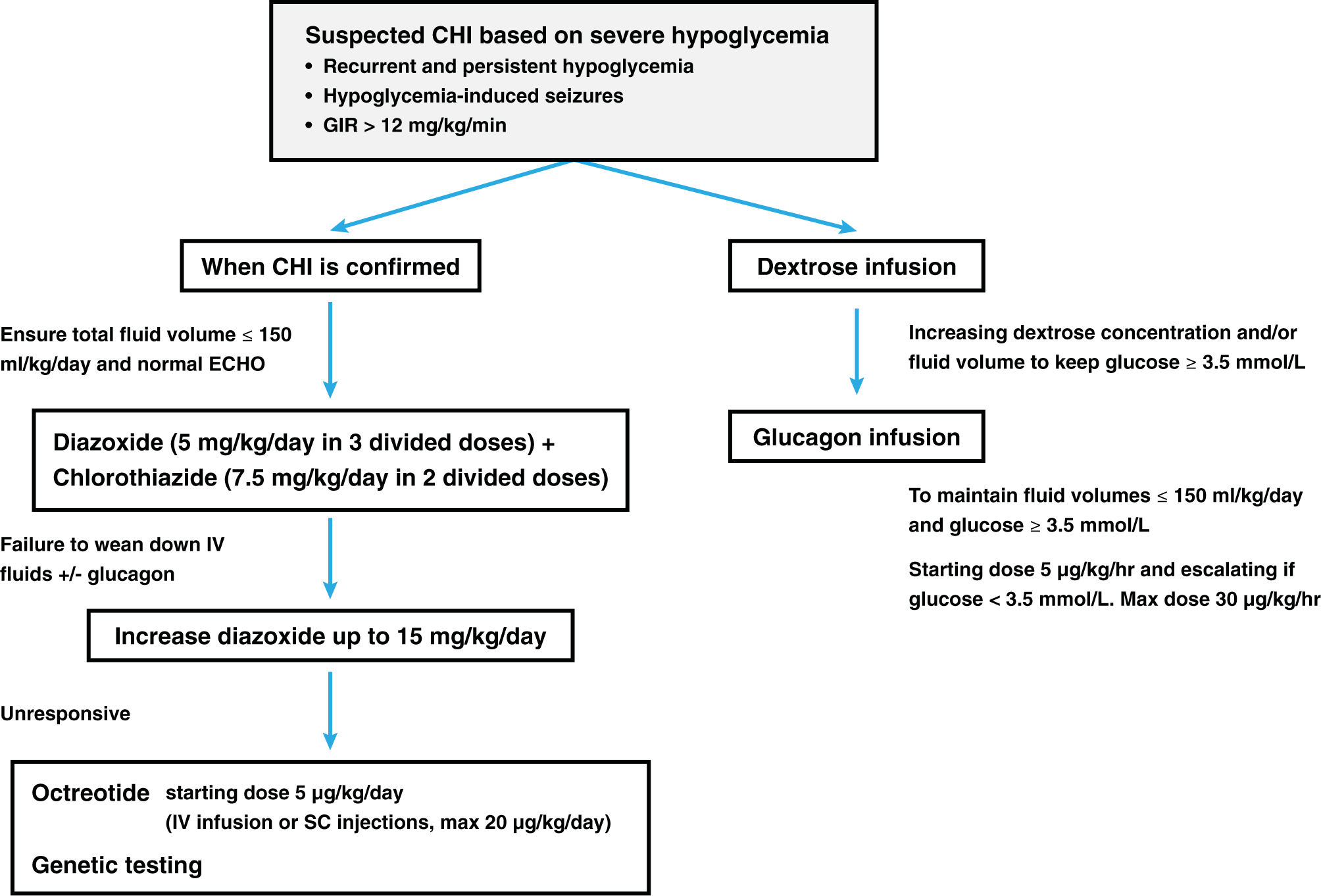

Supplement: Supplemental Figure 1 — Representation of the initial management of patients presenting with severe hypoglycemia in our institution. Dextrose infusion is commenced followed by glucagon infusion in order to maintain glucose concentration ≥ 3.5 mmol/L. When hyperinsulinism is confirmed, CHI-specific medication is initiated and then adjusted according to clinical response. [file Image_1.tif]
